# Prospects for genomic surveillance for selection in schistosome parasites

**DOI:** 10.3389/fepid.2022.932021

**Published:** 2022-09-29

**Authors:** Zachary L. Nikolakis, Richard H. Adams, Kristen J. Wade, Andrea J. Lund, Elizabeth J. Carlton, Todd A. Castoe, David D. Pollock

**Affiliations:** ^1^Department of Biology, University of Texas at Arlington, Arlington, TX, United States; ^2^Department of Biological and Environmental Sciences, Georgia College and State University, Milledgeville, GA, United States; ^3^Department of Biochemistry and Molecular Genetics, University of Colorado School of Medicine, Aurora, CO, United States; ^4^Department of Environmental and Occupational Health, Colorado School of Public Health, University of Colorado, Anschutz, Aurora, CO, United States

**Keywords:** natural selection, selection, schistosomes, population genetics, genomic scans, control efforts

## Abstract

Schistosomiasis is a neglected tropical disease caused by multiple parasitic *Schistosoma* species, and which impacts over 200 million people globally, mainly in low- and middle-income countries. Genomic surveillance to detect evidence for natural selection in schistosome populations represents an emerging and promising approach to identify and interpret schistosome responses to ongoing control efforts or other environmental factors. Here we review how genomic variation is used to detect selection, how these approaches have been applied to schistosomes, and how future studies to detect selection may be improved. We discuss the theory of genomic analyses to detect selection, identify experimental designs for such analyses, and review studies that have applied these approaches to schistosomes. We then consider the biological characteristics of schistosomes that are expected to respond to selection, particularly those that may be impacted by control programs. Examples include drug resistance, host specificity, and life history traits, and we review our current understanding of specific genes that underlie them in schistosomes. We also discuss how inherent features of schistosome reproduction and demography pose substantial challenges for effective identification of these traits and their genomic bases. We conclude by discussing how genomic surveillance for selection should be designed to improve understanding of schistosome biology, and how the parasite changes in response to selection.

## Introduction

Schistosomiasis is a neglected tropical disease caused by schistosome parasites that affects over 200 million people annually, with most infections impacting the health and socio-economic development of populations in Africa, South America, and Asia (1, 2). Schistosomes have two distinct reproductive phases, the first of which includes the pairing of adult male and female worms in definitive mammalian hosts to produce eggs through sexual reproduction, and a second asexual phase which occurs once eggs hatch into miracidia and infect intermediate snail hosts. In the snail host, miracidia become reproducing sporocysts that are eventually emitted as free-swimming cercaria that infect the mammalian host (3). Three *Schistosoma* species are primarily responsible for human infection, and depending on the species, will infect either the urogenital (*S. haematobium*) or intestinal (*S. mansoni, S. japonicum*) tract. Symptoms range from fibrosis of the liver and bladder to anemia and cancer, all with an underlying pathology stemming from immunological responses to egg deposition from sexually mature worms (1, 4). The World Health Organization (WHO) has adopted resolutions calling for regional elimination of schistosomiasis through a multi-pronged approach that includes mass drug administration (MDA) using the anthelminthic drug praziquantel (PZQ), reduction of snail intermediate host populations, and improvements to hydrological and sanitation infrastructure (5).

**Figure 1:**
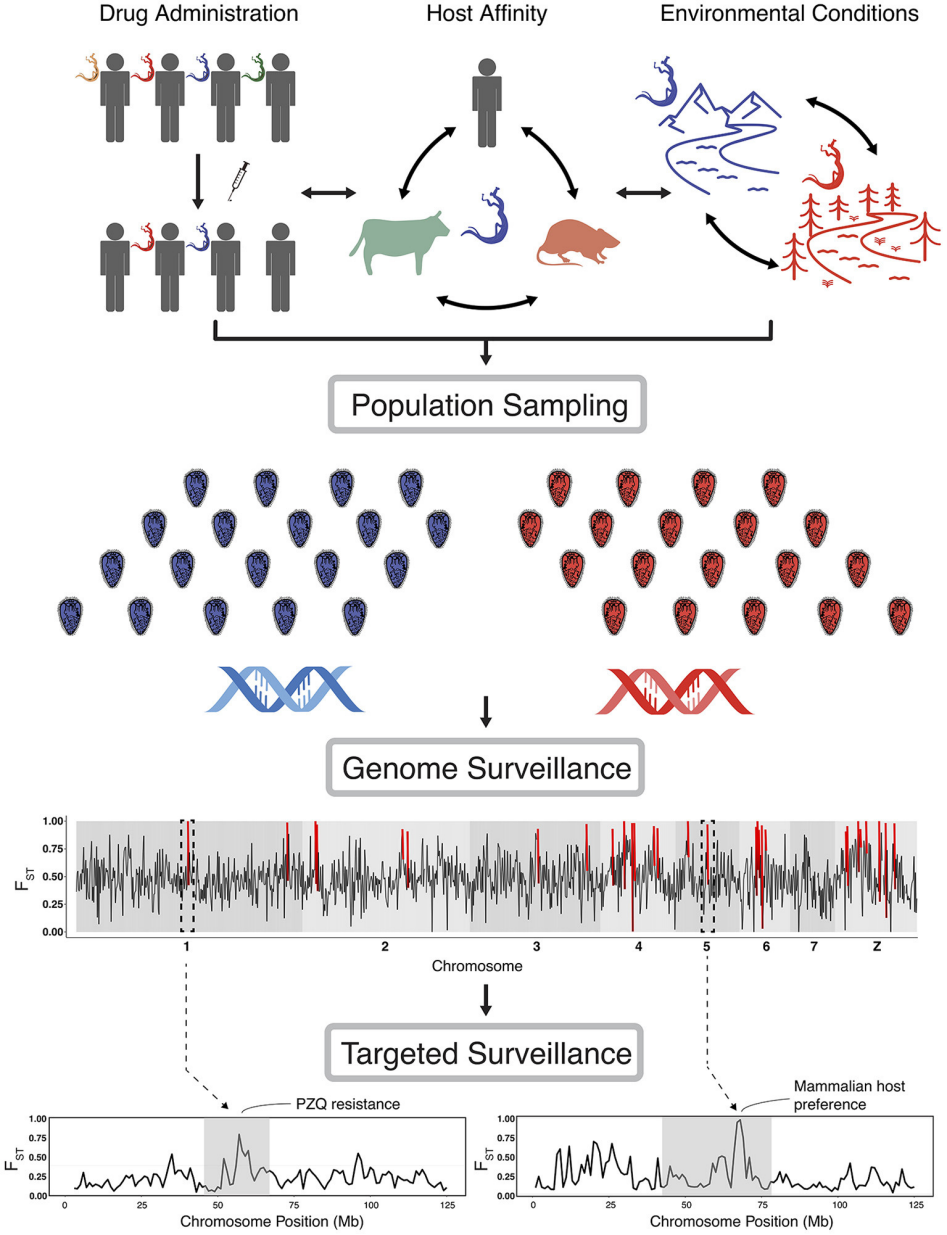
Overview of the implementation of genomic scans of selection. Population sampling is carried out under different scenarios of drug administration (or other control regimes), changes in host affinity, environmental conditions, or simply across a temporal series of population sampling. Genome scans identify targets of selection and inform further study of the genomic basis of relevant traits. Targeted surveillance will then help to assess the presence of alleles and their impact on these traits, and be used to monitor for selection in parasite populations. In genome scan figures (lower graphs) we highlight a hypothetical genomic scan, with red lines indicating loci with extreme values (using *F*_*ST*_ here as an example); dashed lines from particular regions of the genome represent zoomed-in areas of potential interest that could contain genes with known links to epidemiologically-relevant traits (with PZQ resistance and host preference shown as examples). We note that our hypothetical examples of genomic scans use *F*_*ST*_, but such scans could be conducted with a diversity of statistics, which we discuss in the text.

In this context, there are growing concerns that control and elimination practices are creating selective pressures on schistosome populations that may alter schistosome biology, sometimes in ways that could negatively impact current and future control success (6). Moreover, there is substantial evidence that schistosomes respond opportunistically to large environmental change such as dam construction (7, 8). Given these concerns, monitoring how schistosomes respond to diverse environmental contexts represents a valuable and increasingly feasible priority as a component of control strategies (9–11). For example, to surveil, and ideally prevent, the development of drug resistant populations, it is necessary to identify and understand selection pressures and parasite responses when drugs are applied. Other diverse selection-driven responses may include shifts in definitive host preferences, shifts in reproductive behavior, or any other shifts in life history that lead to greater fitness (Figure 1).

In this review we discuss strategies to integrate broad (genome-wide) and targeted (locus-specific) approaches to genomic surveillance for selection across natural populations of *Schistosoma* species. We also update earlier reviews (9), which have discussed the roles of genetic studies for understanding the impacts of control measures on schistosomes, to incorporate modern approaches that leverage whole genome sequencing (WGS) coupled with targeted schistosome sample collection to understand and interpret genetic variation of pathogen populations to guide control programs (12–15). In a recent review, we discussed the ways in which genomic data can be integrated in epidemiological and ecological studies to clarify schistosomiasis transmission pathways (15). In this review, we discuss the theory underlying population genomic scans, review how recent studies have leveraged population-level sampling of genomic variation to conduct genome-wide scans to identify signatures of selection and genes underlying key traits in schistosomes and discuss how future studies can be designed to improve their power and effectiveness. We also review current progress linking epidemiologically relevant traits in schistosomes (e.g., drug resistance, host preference, reproductive output) to their underlying genetic basis, and discuss how future studies and infrastructure may enhance the potential impacts of genomic surveillance for evidence of selection acting on such traits.

## Importance of genomic surveillance for tracking selection in schistosome populations

The world's recent experience with SARS-CoV-2 reinforces the fundamental reality that all natural systems evolve. In the context of infectious diseases and parasites, evolution must be accounted for, monitored, and ideally integrated into control strategies. Understanding how neutral processes of genomic divergence (aka. genetic drift) and natural selection together shape the genomic diversity of pathogen populations is necessary to evaluate how biological systems respond and adapt to their environment, which can ultimately lead to valuable and well-informed predictions to guide strategies for control. For example, control measures may lead to an increase in the frequency of genomic variants that impact drug tolerance, parasite reproductive output, or host specificity. Genomic surveillance to monitor evidence of selection on loci related to these traits may therefore provide early warnings that would enable strategic re-design of control efforts. Furthermore, comparisons of genomic variation between populations, lineages, or temporal population samples can be used to identify regions of the genome that underlie selected traits. Here we argue that longitudinal sampling of natural schistosome populations is a high priority to establish critical references for continued surveillance, with the added benefits that they also may be used to detect evidence of natural selection, population connectivity, and to monitor variation at specific genomic loci associated with traits of epidemiological relevance (e.g., host preference or drug resistance).

## Genomic resources and new approaches enable *Schistosoma* surveillance

All species of *Schistosoma* are diploid and contain 7 autosomes as well as ZW sex chromosomes. Their genomes range from ~390–410 Mbp and tend to be comprised of ~50% readily identifiable repetitive elements (16, 17). Reference genomes for *Schistosoma* species that are responsible for most human schistosomiasis cases [(17–20); *S. mansoni, S. haematobium, and S. japonicum*] now have high-quality chromosome-level reference assemblies (17, 21, 22). These genomes share relatively high degrees of synteny and conserved chromosome numbers (21), and are assembled in relatively large contiguous scaffolds, with scaffold N50 values (defined as the size of the shortest scaffold included in the set of long scaffolds that comprise 50% of the genome assembly) ranging from 4.8 to 48 Mbp. Despite the overall high quality of these genome assemblies, the quality and contiguity of the sex chromosomes in these assemblies are lower, and most references have only included the Z and not the W chromosome. In ZW sex chromosome systems, such as schistosomes, males are the homogametic sex (ZZ) and females are the heterogametic sex (ZW). Most reference genomes are generated from the homogametic sex because this provides equal representations of autosomes and sex chromosomes, and because of the inherent complications of assembling sex chromosomes (e.g., high repeat element content, high heterochromatic content, and relative copy number relative to autosomes) (23–25).

The availability of these reference genomes enables the accurate mapping and interpretation of whole genome sequencing (WGS) data, and thus provides the means to understand how processes such as natural selection, genetic drift, hybridization, and recombination shape genetic diversity in specific schistosome populations. WGS from individual miracidia and cercaria, the readily available schistosome life stages, is challenging due to the low quantity of DNA available from these physically small samples [(15, 26); see also discussion in Rey et al. (6)]. Recent advances that use whole genome amplification (WGA) ameliorate this challenge, and thus substantially increase the feasibility of genomic surveillance by enabling WGS of individual parasites (12, 27, 28). Low-input DNA genomic library preparation approaches that accomplish amplification through PCR have also shown promising results using schistosome eggs or larval stage samples (29).

A number of studies that have used WGS to sequence DNA from miracidia seem to have little host contamination (12, 27, 28), suggesting minimal off-target sequencing of host DNA using filtration and washing protocols to separate miracidia from host stool, as is used for the miracidial hatching test in China (30). However, other studies (13) have reported higher rates of contamination (based on higher fractions of genome sequencing reads that do not map to schistosome reference genomes), suggesting contamination may be more of an issue in some cases. These advances in techniques coupled with decreasing costs of sample preparation and sequencing. For example, at time of publication WGS library prep and Illumina sequencing for a single sample at 20× coverage is ~$300, based on our experience in the context of high parallelization. These recently reduced costs now make it feasible to generate WGS data for many individual samples and to survey patterns within and across populations. Thus, reference genomes and WGS approaches for resequencing genomes from individuals have laid the foundation for a new generation of genomic surveillance programs capable of resolving genetic variation at extremely high precision, and as we discuss below are sufficient to evaluate evidence for selection.

## The theory of genome scans for selection and the delineation of underlying genes

One of the most powerful approaches for genomic surveillance of infectious organisms is to evaluate population genetic variation and “scan” how it changes along the genome. Such genomic scans can dissect evidence that different evolutionary processes have shaped variation differently among loci (31–34), which can track the spread of specific genetic variants (35), monitor genomic changes within and between populations (36), and potentially predict future outbreaks and control efficacy. For a region of the genome, variation can be quantified using population genetic statistics such as the Fixation Index [*F*_*ST*_; (29)], nucleotide diversity [π; (30)], and Tajima's *D* (37). Haplotype-based population statistics were used in scans of schistosome genomic variation (22), including the integrated haplotype score (iHS), which measures extended haplotype homozygosity (EHH) surrounding a given SNP, and compares inferred ancestral and derived alleles (38, 39). Additionally, other haplotype methods such as Identity-By-Descent (IBD) approaches, have been used to infer shared genomic segments between individuals in pathogen populations inherited from a recent common-ancestor, and can be used to show multiple levels of relationships depending on shared segment lengths (40). Genomic scans of schistosome populations (22) have also used cross-population composite likelihood ratio tests [XP-CLR; (31)], which quantify multilocus allele frequency differentiation between populations and account for genetic drift, to test for evidence of selective sweeps. Typically, a battery of such measures are applied [e.g., F_ST_, EHH, XP-CLR; (22, 41, 42)], and the background distribution computed across all genomic regions provides an expectation that can be used to highlight outlier loci with extreme characteristics. For example, abnormally high *F*_*ST*_ differentiaton between regions that differ significantly from the rest of the genome might be of note, as they may represent regions that have experienced strong selection leading to divergence between populations at a particular locus. Genomic regions that harbor outliers may be strong causal candidates for affecting a trait of interest; for example, gene regions affecting drug resistance may be discovered when comparing two or more populations subject to differential drug treatment.

**Figure 2:**
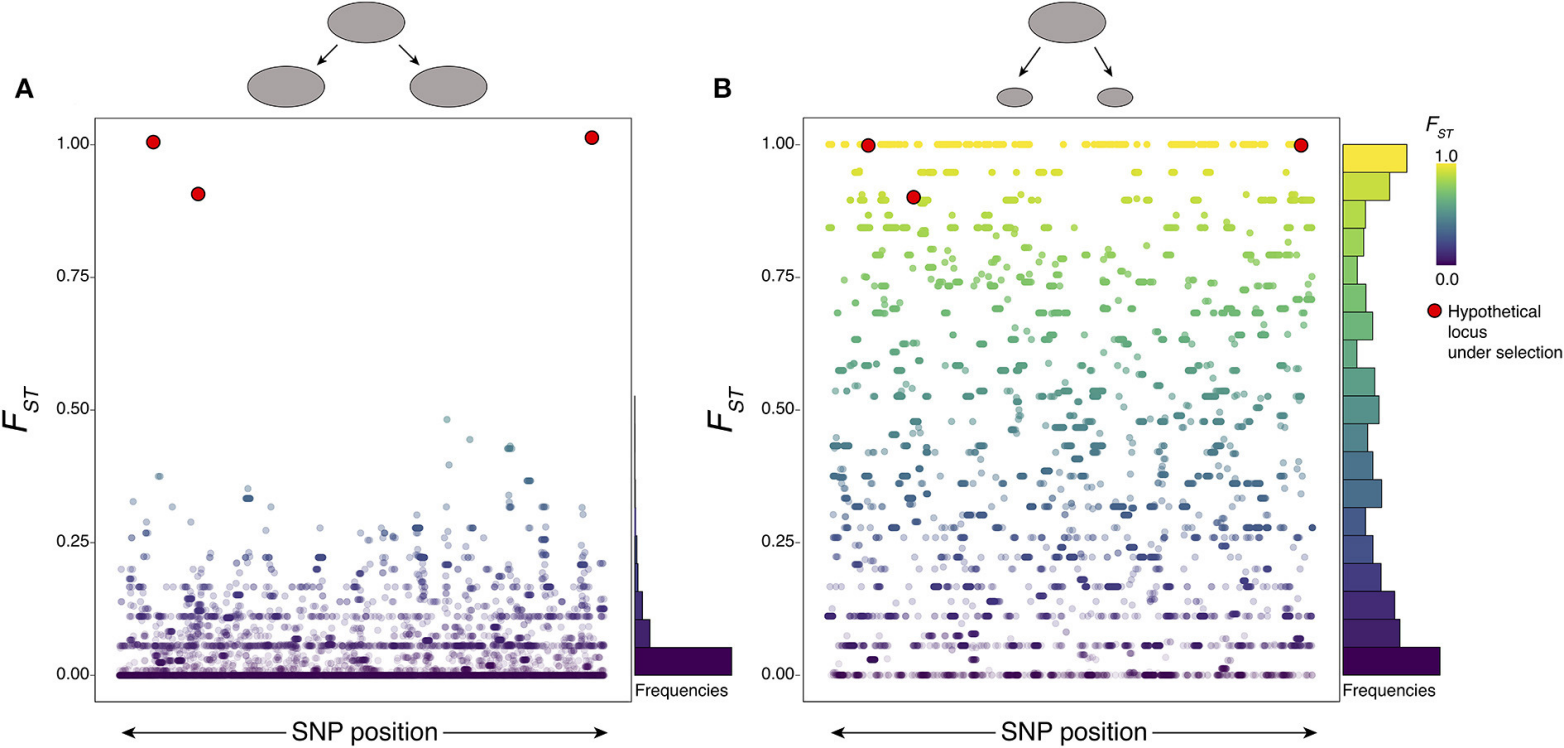
The power and pitfalls of genome scans. Population size affects genetic drift, creating noise in the form of randomly divergent regions of high *F*_*ST*_ that disguise true signatures of selection. Simulated distributions of *F*_*ST*_ are shown (with *F*_*ST*_ at individual loci represented as dots) under two neutral population scenarios indicated by the ellipses on top: **(A)** a moderately large population that split into two similarly sized populations; and **(B)** a similarly sized ancestral population that split into two much smaller populations. In both cases, *F*_*ST*_ values for fixed length genomic regions are colored as indicated by the legend to aid in visual interpretation, and frequency histograms of neutral *F*_*ST*_ values are summarized to the right. The red circles are examples of *F*_*ST*_ values that might be expected for selected loci and are placed with the simulation results to indicate that they would be easy to distinguish in scenario **(A)** but difficult to distinguish in scenario **(B)** due to the large number of high *F*_*ST*_ variants generated under genetic drift alone—indicating high false positive rates regardless of what high *F*_*ST*_ threshold might be chosen. In these simulations, *N*_*e*_ is scaled by the mutation rate μ, with θ = 4*N*_*e*_μ values of 0.1 for the larger populations and θ = 4*N*_*e*_μ values of 0.005 for the smaller populations. These hypothetical examples demonstrate the importance of integrating multiple statistics and approaches to discern evidence of selection to confirm or refute evidence for selection at particular loci, while also illustrating the pitfalls of using single statistical criteria (like high *F*_*ST*_ alone) to make such inferences.

The logic of genome scans is relatively straightforward: population genetics theory predicts that different evolutionary processes will leave particular signatures of variation within and between genomes, and so distinct processes can be inferred if the appropriate signature is observed. Most of the genome is assumed to evolve under random genetic drift, such that the genomic background (e.g., distribution of *F*_*ST*_ among genomic regions) is primarily shaped by demographic processes. A central controlling demographic parameter that impacts patterns of genomic diversity and differentiation is the effective population size (*N*_*e*_), which summarizes the long-term population size effect even when the number of reproducing individuals within a population varies over time. For example, population size bottlenecks (brief periods of greatly reduced population size; Figure 2) tend to reduce genomic diversity, leading to lower average π across all regions compared to a population that maintained large population sizes. Populations that evolve separately will also diverge over time, so that recently separated populations have low differentiation (i.e., lower average *F*_*ST*_ when compared to more well-defined lineages that diverged further in the past (which will have comparatively higher average *F*_*ST*_). In the case of schistosomes, large-scale control efforts that successfully eradicate large numbers of reproductive adults may have such brief bottlenecks, or ideally even dramatic long-term reduction in local *N*_*e*_. Bottlenecks may also be caused by founder effects that occur when a few individuals expand and colonize new environments or hosts. Indeed, previous empirical estimates of N_e_ across multiple *S. mansoni* and *S. japonicum* populations have shown evidence of multiple population bottlenecks within the last 100,000 years. Some inferred bottlenecks appear to be correlated with the timing of human migration into those regions following glacial cycles, and other more recent bottlenecks are potentially linked to modern control efforts (14, 22). These studies have also highlighted differences in *N*_*e*_ among populations within species of schistosomes. For example, *S. mansoni* populations in the Lake Victoria and Lake Albert regions of Africa appear to have experienced recent increases in *N*_*e*_, whereas populations in both West Africa and in regions of the New World show evidence of continuous declines in *N*_*e*_ (14).

Strong natural selection is also predicted to leave striking signatures along the genome, often highly localized perturbations that defy the expectations of drift [(43); Figure 2]. Positive directional selection associated with adaptive traits, also known as a selective sweep, reduces genetic variation in regions of the genome near selected loci (43). This occurs when new mutations or low-frequency alleles are favored, driving them to high frequency and eliminating variation at other nearby regions of the genome that are genetically linked. These allelic sweeps therefore result in regions of the genome with low diversity that are also more differentiated from related populations than other regions that did not experience directional selection. This is detected by observing exceptionally low π and high *F*_*ST*_ compared to the genome-wide background.

Many aspects of schistosome treatment and control may drive selective pressure on the parasites to adapt or die. For the drug treatment example, genetic variants that enable the organisms to resist the drug are more likely to be passed on to the next generation, and over time this will produce the classic signature of a selective sweep. Although the mechanisms may be more complex, life history traits such as timing of cercaria production, or life span of adult worms, for example, may also favor various alleles to sweep through a population. However, our power to detect a selective sweep will depend on the strength of selection that is driving the sweep. Cross-over events during meiosis (recombination events) tend to unlink nearby genomic regions from the selected allele, and there will be more opportunity for recombination events if the sweep takes longer when selection is weaker (or if selection happened longer in the past). Thus, the ability to recognize the signatures of selective sweeps is reduced as time accrues since they occurred, and is also decreased if selection is weaker (44–46).

### Factors that limit power of genome scans

For genomic surveillance of selection on infectious organisms, most genomic scan studies are designed to compare two populations that differ in some key treatment (e.g., presence or absence of MDA) or trait of interest (e.g., host preference). The approach is powerful, but it should be appreciated that some differences among loci may be due to drift alone. Drift is a noisy (stochastic) process, and the genetic linkage that helps identify a selected region also leads to regional levels of linked differentiation due to drift, which makes detection of selection challenging (Figure 2). Such random variation in genetic differentiation among loci will have little to do with the condition or trait of interest. In this context, it is worth appreciating that genome scans can easily be performed on populations without an explicit trait of interest in mind. In that case, they will identify regions of the genome that most strongly differentiate these populations, in many cases regardless of whether selection, drift, or some other process is responsible.

Another important caveat when performing genomic scans is that some divergence between the populations examined may be caused by variation in traits that are not of direct interest. For example, if two populations with different drug treatments are also from distinct environmental conditions, such as different altitudes or aridity, loci may have been selected in response to those environmental differences and not the drug treatment of interest. A basic comparative genomic scan is most powerful for two populations in highly similar environments that are as closely related as possible so that they have diverged as little as possible, thus having few biological differences other than the trait or treatment of interest. This reduces background noise and allows any selective effect due to the trait of interest to definitively stand out.

Genome scans also assume that the trait of interest has a genetic basis, that alleles underlying variation in this trait were under relatively recent and relatively strong selection, and that a reasonable amount of genetic variation had built up in the population prior to selection. If there is little standing variation at alleles that affect the trait, there is little allelic diversity to be reduced by selection and a low ability to differentiate the effects of selection between the two populations. The effect of drift interacts with low levels of standing variation because drift effects are larger in small populations, and population bottlenecks due to control efforts will lower *N*_*e*_, increase drift, and fix unselected alleles between populations.

It is also worth appreciating that selection can take multiple forms that affect diversity patterns (e.g., balancing selection, diversifying selection, negative selection on nearly neutral variants, multiallelic selection), and the interaction of these different forms of selection with drift can complicate or confound interpretation of results. This also means that some forms of selection (e.g., balancing selection) will not be identified by approaches designed to detect other forms of selection, such as scans for selective sweeps (e.g., XP-CLR). Balancing selection may result from highly variable (temporally or spatially) control efforts, and would lead to elevated π, potentially elevated heterozygosity, yet low *F*_*ST*_ between populations; this result is nearly opposite that expected for a selective sweep. Diversifying selection, usually characterized by repeated selection for new alleles at the same locus, might be expected for parasite proteins that are antigenic and selected to repeatedly evade host immunity. Diversifying selection is expected to result in variable (but often low) *F*_*ST*_ and thus may not be detected in scans designed to detect selective sweeps. Polyallelic selection, in which multiple equivalent but divergent alleles are selected at the same time, can also complicate interpretation (47). Due to these challenges, scans of selection should layer multiple methods, inferences, and summary statistics to test whether specific patterns and processes of selection have occurred.

One way to obtain expectations of the effects of selection and drift in various scenarios is to use simulation-based inferences (48–54). Simulations can also provide calibrated expectations about false positive rates and appropriate statistical thresholds for identifying outlier loci in genomic scans. The efficacy of selection is positively correlated with population size (i.e., adaptation is more efficient in larger populations), and simulations can identify genome-wide background expectations under drift. For example, in a small population we expect many loci to have maximal *F*_*ST*_ value (Figure 2B), even in the absence of selection. Simulations can also be beneficial to understand how rapidly adaptation may have occurred in response to control measures (55) and to predict the conditions under which drug resistance may be expected to lead to parasite elimination or to lead to the evolution of drug resistance (56).

## Example workflow for population genomic analyses of selection

**Figure 3:**
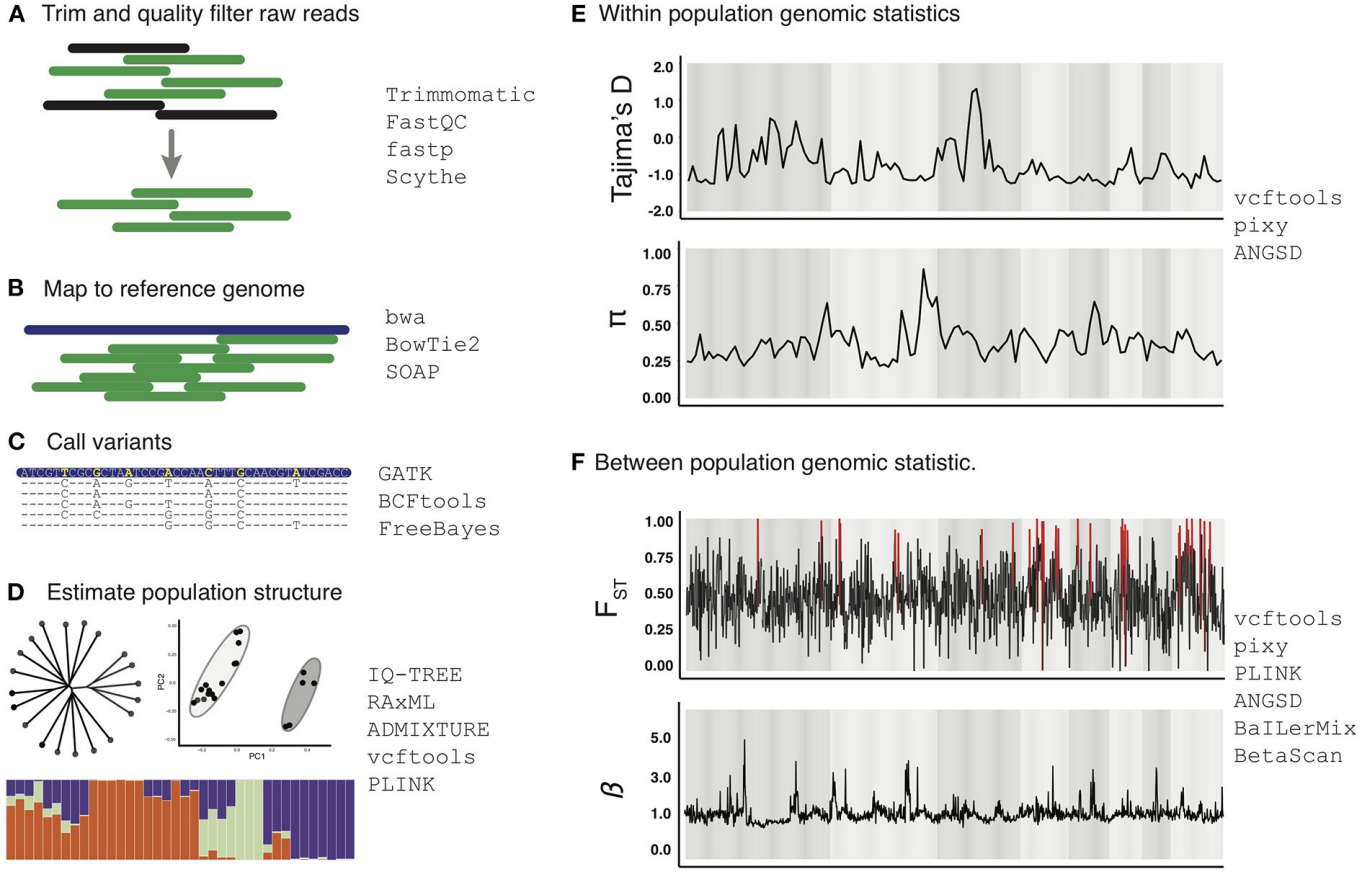
Example work-flow for analysis of whole genome resequencing data for analysis of selection. The example work flow begins with raw genome shotgun sequencing reads that are processed for quality **(A)**, processed reads are then mapped to the reference genome of the respective species **(B)**, and variants within individuals are called **(C)**. Estimates of population structure can then be estimated **(D)**. Within population genomic statistics **(E)** and between population genomic statistics **(F)** can then be estimated in the form of genome-wide scans of these parameters to develop integrated inferences for evidence of patterns of selection in schistosome genomes. All data shown is hypothetical and not necessarily representative of schistosome populations, and are shown only as a graphical representation for illustration purposes. Relevant programs for each step are listed to the right of each panel, and these are cited and discussed in the main text.

In Figure 3 we provide an example workflow for inferring evidence of selection by analyzing population genomic data with existing programs and strategies that can be used in various steps. Standard data analysis of high throughput sequencing data typically starts with processing of raw genome resequencing reads to remove low-quality reads and trim low-quality portions of reads using programs such as Trimmomatic (57), FastQC (https://www.bioinformatics.babraham.ac.uk/projects/fastqc/), fastp (58), and Scythe (59). Processed reads are then mapped to a reference genome using the mapping algorithm bwa, BowTie2 or SOAP (60–62). Sequence variants relative to the reference genome are then called using various approaches such as BCFtools, FreeBayes, or GATK (63–65). Among these, GATK is considered an industry standard because it includes well-established “best-practice” guidelines for multiple variant types (e.g., SNPs and structural variants), which helps maintain transparency and consistency across analyses and studies. It is notable, however, that GATK “best practices” for inferring genomic variants have been designed to interpret human genomic variation, and that it remains an important priority for future work to consider if other modified best-practice approaches may be more appropriate for schistosomes and other parasites. For example, it is possible that expectations related to high levels of inbreeding likely in many schistosome populations may warrant revision of best-practice approaches for minimums of sequencing depth or other parameters associated with inferring genomic variants.

Once genomic variants are inferred, basic inferences of population structure can be estimated using clustering methods to infer population ancestry [e.g., STRUCTURE/ADMIXTURE; (66, 67)], and through discriminant analysis of principal components [e.g., DAPC; (68)], PCA, along with tree-based methods implemented in programs like RAxML (68) and IQTree (69). These initial assessments are important because they provide a first-pass approximation of population structure within the data, which is essential for downstream experimental design considerations, such as analyzing subpopulations separately or filtering highly related individuals to avoid potential stratification issues. Further, population clustering analyses (e.g., PCA, DAPC, STRUCTURE) can also provide insight into levels of connectivity and structure among parasites between hosts, which can provide insight into parasite transmission patterns relevant for informing control measures [see (15) for a detailed review of inferring transmission pathways using genomic data].

There is no single straightforward way to infer evidence of selection. Instead, genomic inferences of selection often require the application of multiple types of genomic scans and integration of these to effectively identify evidence for selection, and to differentiate various modes of selection. The choice of relevant cutoffs for parameters used in genomic scans is often difficult to be certain about; some researchers use confidence intervals, although other more appropriate approaches [e.g., GPPFst; (50)] can be used to predict the null distribution of these parameters under drift alone (without selection), which hold promise for more powerful inferences by calibrating expectations by explicitly considering the demographic history of populations. Because many methods for detecting selection are designed to identify directional selection, such approaches are poorly suited to identify evidence of other forms of selection, such as balancing selection. It is therefore useful to incorporate genomic scans for different modes of selection implemented in programs such as BaILerMix and Betascan (70, 71). Secondary analyses using haplotype-based measures provide additional opportunities to identify evidence for directional selection by detection of selective sweeps using programs like *rehh* (72), which uses methods like extended haplotype homozygosity (EHH). While these methods are important for understanding patterns within empirical data, comparing these measures against simulation-based inferences using programs like SLiM (52) or msprime (73) can also help more appropriately interpret statistics and their expectations under drift vs. selection, thereby improving the accuracy of inferences of selection.

## Prior Schistosome genomic scans for signatures of selection

Multiple studies have examined genomic variation across *Schistosoma* species or populations to test for signatures of selection and thereby predict which loci underlie evidence for selection on divergent traits, or specific traits under study. Crellen et al. (14) used transcontinental sampling within *S. mansoni* to identify evidence of selection between populations by applying codon-based measures of selection in protein-coding regions (i.e., *d*_*N*_*/d*_*S*_) and other approaches that examine ratios of polymorphisms and fixed differences (e.g., Hudson-Kreitman-Aguade and McDonald-Kreitman tests). They identified suites of genes inferred to be under selection, and a subset of these loci were associated with functional categories of genes involved in host-parasite interactions and genes required for mammalian-host infection. Li et al. (74) PCR-amplified and sequenced multiple candidate genes across populations of *S. japonicum* from China, comparing *S. japonicum* from lake vs. mountainous regions, and used population genetic summary statistics (e.g., Tajima's D, π, Fu's, *F*_*ST*_) to identify evidence of divergent selection on genes that encode tegument-associated antigens (74). These proteins are thought to be involved in intermediate snail host immunity, and evidence for selection acting on these genes was presumed to be linked to divergence between lineages of *Oncomelania* snail hosts in mountainous and lowland regions.

Other studies inferring patterns of selection used WGS data to compare genomic variation across either temporal or experimental treatments. Berger et al. (13) sampled populations of *S. mansoni* from school-aged children in Uganda to identify loci putatively under selection following drug administration by using a combination of haplotype measures (i.e., iHS and XP-EHH), relative and absolute population differentiation (*D*_*XY*_ and *F*_*ST*_), and π and accounted for the impacts of linkage disequilibrium and close-order relatedness among samples. They identified significant variation between populations across multiple genomic regions, with the highest occurring on chromosome two that contained loci coding for sodium/potassium/calcium ion exchanger proteins.

Some studies followed up genomic scans with secondary experiments involving gene expression analyses or knockdown/interference studies. Le Clec'h et al. (75), for example, identified a candidate locus that may underlie variation in drug response by performing genome-wide association (GWAS) analyses between laboratory-bred strains of *S. mansoni* that showed significant differences in survival between high and low doses of PZQ. They identified several variants (single nucleotide polymorphisms and structural variants) that were associated with PZQ response and functionally validated these by comparing gene expression differences between lab and natural populations. Additionally, they identified natural populations possessing these alleles that impact drug-response by analyzing previously published exome sequence data from *S. mansoni* populations in Africa, South America, and the Middle East. Luo et al. (22) generated a new chromosome-level reference genome for *S. japonicum* and used this to interpret additional WGS data to infer selection patterns in natural populations. They used multiple genome wide scans to identify regions with signatures of selective sweeps and focused on candidate loci that are involved in both intermediate and definitive host-affinity pathways. They functionally validated one candidate gene for definitive host preference (*GATAD2A*) using RNAi methods and demonstrated how decreased expression of this gene leads to abnormal development of reproductive organs in sexually mature worms and shifts in differential mammalian host infection outcomes. In a separate genome scan between *S. japonicum* populations they identified another locus (*Lmln*) with strong signatures of selection potentially related to intermediate host preference and showed that this gene is expressed in schistosome life stages important for intermediate host infection. Further supporting these inferences for the role of *Lmln* in *S. japonicum*, a separate study also linked *Lmln* to infection stability for intermediate snail hosts in *S. mansoni* (76).

These studies collectively establish precedence for leveraging genomic scans of selection to identify candidate loci for important traits in schistosomes. However, many of these early studies also suffered from reduced statistical power due to relatively limited sampling of populations, potential issues stemming from population stratification and the highly inbred nature of sampled populations, and to some extent from the existence of population structure between compared populations (44). Indeed, many of these analyses identified hundreds of potential candidate genes, which presumably include large fractions of spurious candidates, making it logistically difficult to investigate them all experimentally (38, 44).

### Experimental designs for more powerful genome scans to identify selection

Because of the limitations of previous pioneering studies, it is relevant to consider how population genomic analyses can be designed to most powerfully leverage their ability to identify important patterns of selection linked to control efforts and use these inferences to further identify genes underlying relevant traits. To effectively account for drift when conducting genomic surveillance of schistosomes, we need accurate and reliable estimates of complex demographic histories that likely include changes in effective population size (i.e., bottlenecks, founder effects), substructure, migration, and other demographic factors, which is not always an easy task (77). Demographic estimates that are fit directly to the study populations (i.e., specific *N*_*e*_ estimates for each population) can be used to calibrate the null expectations of drift in a population-specific manner [e.g., (32, 78–81)]. Bayesian predictive methods (e.g., approximate Bayesian computation and posterior predictive methods) explicitly incorporate demographic estimates while accounting for uncertainty in these estimates to simulate genomic data under drift, which can be compared to the observed data (49, 81–83). Some methods, like the XP-CLR approach, are designed to inherently account for drift while identifying selective sweeps, and are also more powerful for identifying more ancient selective sweeps (48). Further advances in incorporating the impacts of drift through demographic modeling and simulation also hold great promise and may improve predictive approaches with more realistic models designed to incorporate the true demographic history of populations. Recently, a number of machine learning applications of artificial neural networks [i.e., artificial intelligence (AI)] have been developed to predict targets of selection with high accuracy and robustness, which may prove fruitful for genomic surveillance of schistosomes (84), including demonstrations for predicting selection in malaria parasites with genome scans (85). Indeed, AI approaches are rapidly gaining traction in a variety of population genetic applications (86), including prediction of selection (87), migration (86), recombination (88), demography (81), population origins (89), and other factors relevant to schistosome biology and control.

The biology of schistosomes requires particular attention when considering experimental design for genomic scan approaches to detect and interpret patterns of selection. Schistosomes are overall genetically diverse, but they can be far more closely related in small geographic areas such as in areas of re-emergence or hotspots (6, 12, 27, 90). This makes sense biologically because of the large number of eggs a single worm pair can produce, combined with the clonal nature and large numbers of cercaria produced from a single snail intermediate host. Even in an area in which schistosomes have been nearly eliminated, a single introduction event into snail habitat from an infected definitive host, for example, can lead to rapid expansion of highly-related schistosome infections in a geographically proximate population of definitive hosts. From a genetic standpoint, this can lead to inbreeding and population bottlenecks in such schistosome populations that make it more difficult to identify genomic regions that are the principal targets of selection from those regions not under selection (e.g., Figure 2). Elevated levels of genetic relatedness within populations (6, 12, 27, 90) defy default assumptions of many population genetic inference approaches and substantially reduce the power of scans for selection. To overcome this and increase the power and accuracy of future genomic scan studies, experimental designs for collection and sequencing of samples for studying selection should strategically incorporate larger sample sizes that minimize inclusion of closely related individuals, yet also minimizes the divergence and population structure between populations compared. Alternatively, with larger sample sizes, genetic structure can be identified and accounted for by conducting independent analyses within sub-populations. Because relationships among miracidia are not well-known ahead of time, initial sampling should be large enough that highly related samples can be removed for some analyses, and still be sufficient to accurately estimate population allele frequencies. One example of a strategy that would minimize these pitfalls is to obtain a time series of population genomic sampling (still with a large sample of individual parasites and excluding close relatives). Because inferences of selection fundamentally depend upon accurate estimation of population allele frequencies, greater sampling of unrelated individuals, compared to prior studies, is a central goal for future more powerful and accurate inferences of selection.

## Review of candidate genes for targeted genomic surveillance in schistosomes

### Prospects for targeted surveillance using genome scans for selection

Through genomic surveillance of genetic variation at candidate loci known to underlie key epidemiologically-relevant traits (e.g., drug susceptibility, host specificity, parasite reproductive output), researchers can track the geographic distribution of specific genomic variants at loci with putatively known functional ramifications, providing them with insight to guide control strategies. This approach, for example, has been effective to understand the population dynamics and spread of drug-resistance alleles in *Plasmodium spp*. and insecticide-resistant genotypes in the *Anopheles spp*. vectors across Central Africa (91–93). These approaches largely depend upon knowledge of the functional and phenotypic ramifications of genes underlying key life history traits, where variation among loci may contribute to potential variation in drug response or other factors that impact parasite fitness (91, 94–96). Recently, however, researchers have begun to identify candidate loci in schistosomes associated with traits of interest that would enable more direct interpretations of genetic variation from schistosome populations *via* genomic surveillance (14, 97). Currently, there are three major categories of candidate genes that underlie critical traits relevant to the control of human schistosomiasis: (i) drug-resistant loci, (ii) host-shift related loci, and (iii) loci related to parasite life history traits. Here we discuss these candidate loci and the potential to develop additional links between functionally relevant genes and schistosome biology that would identify more candidate loci. While these candidate loci provide some ability to interpret patterns of selection to parasite traits they may be associated with, we argue that future studies of selection may substantially expand and refine this set, and even the identification of selection without *a priori* understanding of the function of loci it effects represents important information for understanding impacts of control efforts.

### Genes linked to drug resistance in schistosomes

Most cases of schistosomiasis are treated with praziquantel (PZQ), with oxamaniquine (OXA) being used historically in limited populations (98). While evidence for the reduced efficacy of PZQ has been documented in natural populations (99–105); see also Cioli et al. (106) for a review on this, the potential for PZQ-resistance to emerge has been demonstrated multiple times in laboratory settings (75, 106). These laboratory-based studies found evidence that schistosomes can evolve variation in response to drug treatment after only a few generations of strong selection, but tend to reduce the fitness of the parasites in other ways (75, 106). This indicates that such traits may evolve rapidly in nature if selection is strong and consistent enough, or the organisms find compensatory mechanisms around the detrimental effects of initial drug resistance strategies. A previous study also provided new evidence for a specific genetic locus that may underlie variation in drug response in *S. mansoni* (75). Also, the diversity of distinct mechanisms (and distinct loci) that may contribute to drug response or resistance remains an open question (107, 108).

Indeed, drug resistance or tolerance alleles may evolve through novel allelic variants (i.e., *de novo* mutations), or may already be present as standing variation in low frequencies in natural populations. Because existing variants could expand rapidly under strong selection from control efforts, monitoring populations for variation in loci linked to drug resistance is particularly important. Previous research identified the underlying locus and variant linked to OXA resistance in *S. mansoni*, a loss of function mutation within a sulfotransferase (107). Additional studies showed that that OXA-resistant alleles are widespread in natural *S. mansoni* populations, and appear to have existed prior to the use of this drug (97); these findings also refuted prior inferences that resistance had evolved in the 1970's, which subsequently led to the discontinuation of OXA as a first line treatment (107). Other laboratory studies showed substantial differences in the effectiveness of OXA across *Schistosoma* species, being most effective for *S. mansoni* but less effective for species such as *S. haematobium* and *S. japonicum* (108).

In contrast to progress understanding mechanisms of OXA resistance, the precise molecular targets and mechanisms of action of PZQ are not entirely clear (107), although a suite of candidate loci and molecular mechanisms associated with PZQ activity and resistance have been proposed (75, 105, 109, 110). Candidate genes related to PZQ susceptibility or resistance that have been proposed fall into several categories: (i) genes associated broadly with drug resistance [e.g., cytochrome p450s, ABC multidrug transporters (109, 111–114)], (ii) genes plausibly linked to PZQ-specific effects on schistosomes, including genes affecting calcium transport and muscle function [e.g., *CamKII*, calcium channel subunits, *p2X* receptors, *regulatory myosin light chain*; (111, 112, 115–117)], and adenosine uptake [e.g., adenosine receptor *ador1, SLC29* nucleoside transporters (116, 118)], (iii) genes related to reproduction (e.g., *p14* eggshell protein gene, *CPEB1*), and (iv) genes related to growth and development [e.g., TGFβ, EGFR pathways; (119, 120)]. A set of previous studies showed compelling evidence that PZQ resistance in *S. mansoni* could be mediated by the gene *TPRM*_*PZQ*_, which encodes an ion channel that binds PZQ (75, 120). Variation in this gene and an adjacent genomic region correlates with PZQ resistance in lab strains, and PZQ resistance may be related to *TPRM*_*PZQ*_ gene expression (75).

Expanded monitoring of genomic variation at these candidate loci, and further identification of genes selected following intense control efforts, are key priorities to improve our understanding of the mode of action and specific targets of PZQ. Genotypes that persist or increase in frequency following drug treatments may provide early warnings for drug resistance and improve our understanding of the relevant loci and molecular mechanisms.

### Genes associated with host specificity and host shifts

Schistosomes likely adapt to different host environments, and selection for genomic variation related to host-specificity is a critical target for genomic surveillance for selection. *S. japonicum* can infect multiple mammalian hosts (121) and the relative importance of different hosts varies by regional context (122, 123). Recent evidence also suggests that hybridization and introgression of different schistosome species [recently reviewed in Rey et al. (6)] may act as a source for the introduction of allelic variants that impact host specificity (124). For example, previous studies have documented the occurrence of hybridization between ruminant- and human-infecting species of schistosomes (*S. haematobium/curassoni* and *S. haematobium/S. bovis)* infecting children in Senegal, which may have the potential to facilitate shifts in host affinities (125). These studies collectively highlight the importance of genomic surveillance that may, for example, be capable of detecting evidence of selection on genomic variants, derived from hybridization events that introduce new alleles, which may enable rapid shifts in host preference.

A recent study used population and functional genomic approaches to identify candidate loci involved in both intermediate and definitive host-affinity, and found strong signals of selection across endemic populations of *S. japonicum* (22). One candidate gene, *GATAD2A*, was shown to be integral in the development of reproductive organs in sexually mature schistosomes, indicating that variation in this locus may dictate definitive host pathogenicity. Additionally, an outlier locus *Lmln* was shown to be significantly upregulated in the miracidia, the life stage that infects the intermediate snail host (22). This study also identified many other candidate genes potentially involved in host preference, suggesting that these traits may be highly polygenic, and that the potential is great for future studies to further refine candidate genes associated with host preference. Beyond studies of host preference, understanding the relationships between human-host outcomes and parasite genotypes may help identify parasite genotypes that correlate with host pathologies or symptom severity. Such studies, however, would be complicated by multiple infections of schistosomes per host, which in some cases includes a diversity of genotypes per host (13).

### Loci associated with schistosome life history and control efforts: Largely the unknown

Many other life history features of schistosomes presumably have a genetic basis and would be important to monitor through surveillance for evidence of selection. The mechanisms producing such traits may be largely unknown, and the challenge for the field is to identify loci that underlie these traits. Schistosomes may alter life history traits such as reproductive output, longevity, and cercarial emergence timing to better suit constraints imposed by control measures or environmental conditions. For example, one potential interest for genomic surveillance includes loci associated with reproductive output, but our understanding of candidate loci for these traits is limited (119, 126). However, a previous study identified multiple genes that show sex-specific expression differences, with many loci underlying overall reproductive output from female schistosome parasites (127). Another study identified the gene encoding the enzyme β-alanyl-tryptamine that is produced by mature male schistosome parasites and is responsible for inducing female reproductive development (128). Cercarial emergence time is another relevant life history trait that is likely related to host shifts but has not been linked to candidate loci. Cercariae tend to emerge from snails during periods that optimize contact with specific mammalian hosts (129, 130), and there is evidence that this trait varies across populations and species (131–133). Continued progress understanding the genomic loci that underlie life history traits would be valuable for targeted surveillance and may be discovered through well-designed population genomic comparisons to detect evidence of selection associated with variation in these traits.

Beyond identifying loci underlying *a priori* traits of interest, monitoring for and identifying any genomic signatures of selection in schistosome populations could provide new perspectives on parasite biology, some of which may be otherwise difficult to predict. Even if we do not currently understand the phenotypic or functional relevance of genes under selection, selection reflects adaptive changes to parasite biology that improve their survival and fitness. Identification of selection in naïve *a posteriori* analyses would still provide the community with loci to prioritize for experimental studies and may lead to unexpected insight into the factors that promote schistosome survival and persistence, which is ultimately central to the design of effective control and elimination programs.

## Conclusions and future directions

We argue that population genomic studies to identify evidence of selection on schistosome populations can play a transformational role in the development of strategies to reduce the global burden of human schistosomiasis. The promise of this approach is supported by the substantial insights provided by recent studies that use these approaches. Genomic data sampled at regular intervals globally can be used in a comparative context to understand shifts in parasite population dynamics, patterns of selection, and responses to control efforts, and greater efforts to collect and share these data would expedite progress. Also, further improvements in experimental design for genomic studies of selection, including more strategic sampling of *Schistosoma* populations over time, under different types of control measures, among definitive host species, and targeting independently replicated selection events, would provide greater power to estimate the specific strength and direction of selection at specific genes or loci (134). Lastly, continued efforts to link selected patterns of schistosome genomic variation to key life history traits of epidemiological relevance (e.g., drug resistance, cercarial emergence times, parasite reproductive output) are a priority for increasing the interpretive potential and ultimate value of genomic surveillance for selection.

Reductions in the cost of genomic sequencing, and increasing amounts of information that links epidemiologically-relevant traits in schistosomes to their genomic bases, has led to the increasing value and feasibility of genomic surveillance for informing control efforts [see Lund et al. (15) for recent review on this topic, including tradeoffs associated with genome-scale data collection]. We argue that genomic surveillance, both for any evidence of natural selection genome-wide, or for evidence of new variants at well-characterized loci linked to relevant traits, provide complementary information for guiding public health officials on impacts of control measures and their efficacy. Central to the success of such surveillance programs, it is critical that monitoring programs begin conducting regular longitudinal sampling of populations (at least sample collection and storage, if not sequencing) that would provide the necessary foundation for comparative analyses. Progress across all these areas would further advance the capacity for genomic surveillance for selection to impact strategic control effort development and facilitate elimination goals globally.

## Author contributions

DP and TC contributed to the conceptualization, planning, writing, and editing of this review. ZN contributed fundamental writing and figures and edited the review. RA contributed a figure and contributed to writing. KW, AL, and EC contributed some writing and editing. All authors contributed to the article and approved the submitted version.

## Funding

Support was provided by a National Institutes of Health (NIH) grant 1RO1AI134673 to EC, TC, and DP.

## Conflict of interest

The authors declare that the research was conducted in the absence of any commercial or financial relationships that could be construed as a potential conflict of interest.

## Publisher's note

All claims expressed in this article are solely those of the authors and do not necessarily represent those of their affiliated organizations, or those of the publisher, the editors and the reviewers. Any product that may be evaluated in this article, or claim that may be made by its manufacturer, is not guaranteed or endorsed by the publisher.
